# Changes in fatigue, barriers, and predictors towards physical activity in advanced cancer patients over a period of 12 months—a comparative study

**DOI:** 10.1007/s00520-021-06020-3

**Published:** 2021-02-19

**Authors:** J. Frikkel, M. Beckmann, N. De Lazzari, M. Götte, S. Kasper, J. Hense, M. Schuler, M. Teufel, M. Tewes

**Affiliations:** 1 West German Cancer Center Essen, Department of Medical Oncology, University Hospital Essen, 45147 Essen, Germany; 2 Department of Psychosomatic Medicine and Psychotherapy, University of Duisburg-Essen, LVR-Klinikum Essen, 45147 Essen, Germany; 3 Department of Pediatric Hematology/Oncology, Center for Child and Adolescent Medicine, University Hospital Essen, Essen, Germany; 4 German Cancer Consortium (DKTK), Partner Site University Hospital Essen, 45147 Essen, Germany

**Keywords:** Fatigue, Depression, Motivation, Barriers, Physical activity, Advanced cancer patients

## Abstract

**Purpose:**

Physical activity (PA) is recommended to improve advanced cancer patients’ (ACP) physical functioning, fatigue, and quality of life. Yet, little is known about ACPs’ attitude towards PA and its influence on fatigue and depressiveness over a longer period. This prospective, non-interventional cohort study examined ACPs’ fatigue, depression, motivation, and barriers towards PA before and after 12 months of treatment among ACP

**Methods:**

Outpatients with incurable cancer receiving treatment at a German Comprehensive Cancer Center reporting moderate/severe weakness/tiredness during self-assessment via MIDOS II were enrolled. Fatigue (FACT-F), depression (PHQ-8), cancer-related parameters, self-assessed PA behavior, motivation for and barriers against PA were evaluated (T0). Follow-up data was acquired after 12 months (T1) using the same questionnaire.

**Results:**

At follow-up, fatigue (*p*=0.017) and depressiveness (*p*=0.015) had increased in clinical relevant extent. Physically active ACP did not show significant progress of FACT-F (*p=0.*836) or PHQ-8 (*p=*0.799). Patient-reported barriers towards PA remained stable. Logistic regression analyses identified *motivation* as a positive predictor for PA at both time points (T0, *β*=2.152, *p*=0.017; T1, *β* =2.264, *p*=0.009). *Clinically relevant depression* was a negative predictor for PA at T0 and T1 (T0, *β*=−3.187, *p*=0.044; T1, *β*=−3.521, *p*=0.041).

**Conclusion:**

Our findings emphasize the importance of psychological conditions in physical activity behavior of ACP. Since psychological conditions seem to worsen over time, early integration of treatment is necessary. By combining therapy approaches of cognitive behavioral therapy and exercise in interdisciplinary care programs, the two treatment options might reinforce each other and sustainably improve ACPs’ fatigue, physical functioning, and QoL.

**Trial registration:**

German Register of Clinical Trials, DRKS00012514, registration date: 30.05.2017

## Introduction

Therapy improvements achieved by cancer research and early integration of palliative and supportive care lead to a longer survival period in patients with incurable cancer (advanced cancer patients; ACP) [1, 2]. As a result, the quality of life of patients and the factors influencing it are increasingly moving into the focus of optimized care. Especially, cancer-related fatigue (CRF) is one of the most distressing symptoms in ACP [3, 4]. The presence of weakness and tiredness cannot be improved by rest and is more agonizing than sleepiness experienced by healthy individuals, which indicates the syndrome of CRF [5–7]. The National Comprehensive Cancer Network guidelines [5] recommend several non-pharmacological interventions including physical activity and psychosocial therapies as treatment of CRF. Several studies and meta-analyses showed a promising way through exercise to reduce fatigue and improve ACPs’ physical functioning and maintain their independence [5, 8–11]. Recently published data [12] suggests cognitive behavioral therapy as a helpful treatment approach for the same purpose. The American Cancer Society’s latest guidelines [13] recommend exercise for ACP adjusted to their individual physical abilities. In contrast, only less than 30% manage to be physically active at all [14–16]. While there is some knowledge regarding cancer survivor’s barriers of physical activity [17–21], only little is known about barriers towards exercise in ACP. Studies [14, 22–24] examined barriers towards physical activity among ACP or a mixed cohort of cancer survivors and ACP. They found lack of motivation, active systemic cancer treatment and its side effects, overwhelming fatigue, and psychosocial factors (e.g., depression, no access to facilities, bad weather) as possible reasons for patient’s inactivity. In addition to these qualitative studies, supplementary quantitative data would help to improve the current state of research. Furthermore, data about fatigue and its accompanying symptoms in ACP or cancer survivors for a longer duration of time is scarce. Few surveys indicate that CRF might increase over time [25–28]. Since there is a steady impact of cancer therapy on patients’ quality of life [29], more information on changes in barriers related to cancer treatment might help in developing suitable exercise programs. The study objective was to compare self-reported fatigue, depression, motivation, and barriers to exercise before and after 12 months of cancer treatment among ACPs. As secondary objective, we aimed to explore the differences in predictors of self-assessed physical activity between before and after 1 year.

## Methods

### Study protocol and patient recruitment

The study was conducted in a large outpatient unit of a German Comprehensive Cancer Center and had a prospective, non-interventional design. All outpatients of the cancer center quarterly answered a validated self-assessment instrument (MIDOS II (the new minimal documentation system) [30]) for the purpose of measuring ACPs’ physical and cognitive symptom burden. The MIDOS II is a German variation of the Edmonton Symptom Assessment Scale (ESAS) [30]. Symptoms are described in a 4-point Likert scale with following options: none, low, moderate, and severe. Patients suffering from moderate to severe tiredness and/or weakness were regarded as eligible for the study. Additionally, participants had to meet the following inclusion criteria: age above 18 years, histologically confirmed cancer with UICC stage IV, capability to understand and answer the German questionnaire, and absence of serious pulmonic or cardiac comorbidities.

Patients fulfilling the eligibility criteria and agreed to participate obtained an information sheet. An in-person paper-based questionnaire was completed on the same day of signing the consent form (T0). All patients underwent some kind of cancer treatment during T0. After 12 months, the paper-based follow-up questionnaire was sent by mail and patients were asked to return the completed questionnaire at their next appointment (T1).

### Questionnaire design

The applied questionnaire consisted of 64 items including the Functional Assessment of Cancer Therapy Fatigue (FACT-F, [31–34]); the Patient Health Questionnaire depression scale (PHQ8, 8 items [35–37]); demographic data (6 items); anticipated psychological, physical, and social barriers (5-, 12-, and 8 items); and questions regarding participants’ physical activity behavior (6 items). Detailed information on the questionnaire structure has been published previously [14].

The FACT-F is a 13-item questionnaire measuring self-reported fatigue. Therefore, a 5-point Likert scale is used. Scores range from 0 (high symptom burden) to 52 (low symptom burden), with ≤ 34 points indicating a pathological FACT-F Score [34]. The PHQ8 is a common tool for measurement of depressiveness. It is based on 8 items with a score of 0 to 3 points. The PHQ-8 score ranges from 0 to 24 points, a score ≥ 10 points to clinically relevant depression [36].

For information on demographics, patients were asked for marital status, living situation, number of children nationality, and educational attainment.

Patient-reported motivation towards physical activity, their activity before cancer diagnosis, their interest in attending an exercise program, and their knowledge about exercise and QoL were measured on a 5-point Likert scale (0= not at all; 1= a little bit; 2= somewhat; 3= quite a bit; 4= very much) [17, 38, 39]. For calculation of relative risk, the answers “not at all”/”a little bit” and “somewhat”/“quite a bit”/“very much” of the 5-pont Likert scale were combined.

To assess subjective physical activity, patients could cross mark their current activity level and add frequency (1= 1 time; 2= 2–3 times; 3= more than 3 times per week) and intensity (1=light, 2=moderate, 2= severe); or in case they have a workout partner, whether their life partner was working out and if they were participants in an exercise program.

For evaluation of anticipated physical barriers in ACP [17, 18, 39], several somatic symptoms (weakness, pain, shortness of breath, tiredness, vomiting/nausea, and joint complaints) were selectable. Furthermore, patients could answer “yes” or “no” questions regarding if they felt weakened due to cancer therapy, had been hospitalized frequently, are afraid of damage from exercising, and if their still smoking.

For assessment of possible social barriers [24, 39], patients could choose between “no local physiotherapist,” “no payed transport,” “missing prescription,” “lack of time,” “stressful daily life,” “too many other commitments,” “bad weather,” and “PA is too expensive.” If applicable, multiple selections of social barriers were possible.

### Patient-related data

Patient-related data including gender, age, comorbidities, tumor entity, type of cancer treatment, previous palliative therapy lines, disease progress over time, and performance status were assessed by patient file revision.

### Statistical methods

**Fig. 1:**
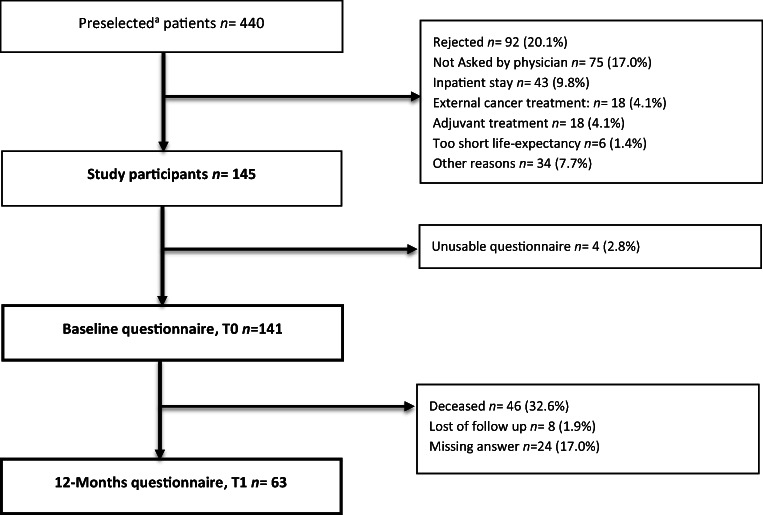
Flowchart of patient enrollment^a^. For preselection the validated MIDOS II [31] was used; patients indicating moderate to severe tiredness/weakness were considered as eligible

**Fig. 2:**
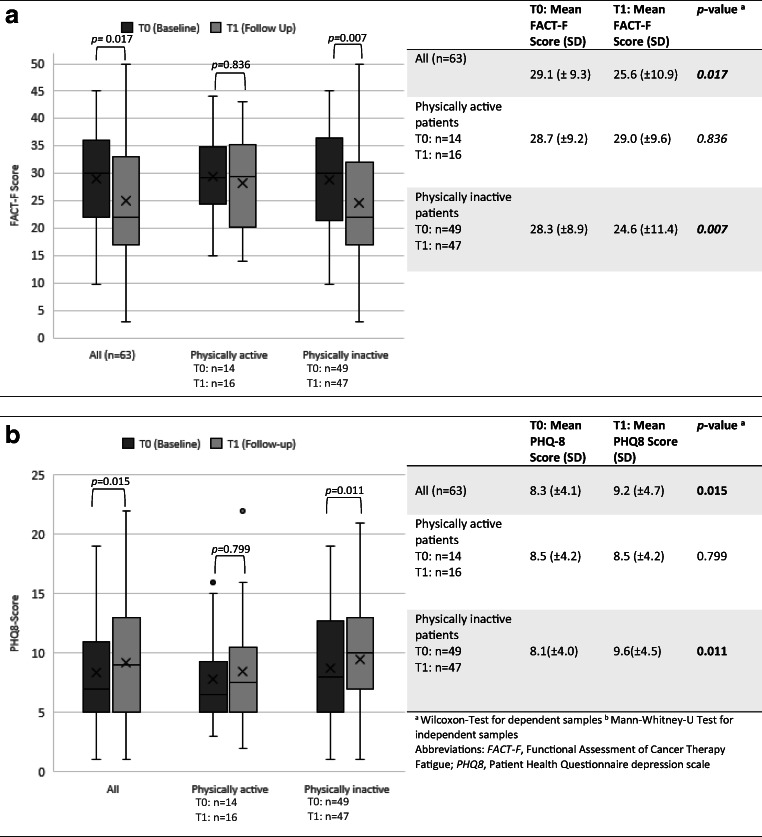
**a** Comparison in means of cancer-related fatigue at baseline (T0) and after 12 months (T1)**. b** Comparison of depressiveness at baseline (T0) and after 12 months (T1)

For descriptive statistics presented in Table 1 and Fig. 1, median, mean values, and standard deviations were generated by SPSS (Version 23). For group comparisons, between the study population at baseline (T0, *n*=63) and after 12 months (T1, *n*=63) shown in Table 1, we used the Fisher’s exact test on nominally scaled variables. For comparison in means, we used the paired *t* test on normally distributed variables, while the non-parametric Wilcoxon test was used on non-normally distributed variables. For comparison of means in fatigue, depression, and motivation at baseline and after 12 months, as well as group comparisons between physically active and physically inactive patients at T0 and T1 (Fig. 2), the non-parametric Wilcoxon test was used. Further group comparisons (e.g., physically active vs. physically inactive patients) were performed depending on sample sizes and scale level (Fisher’s exact test*, χ*^2^-Test, Mann-Whitney *U* Test, independent *t* test). In order to measure the strength of relation between barriers towards exercise at baseline and after 12 months (Table 2), relative risks (RR) and the 95% confidential interval for each barrier were calculated. Fisher’s exact test was used for calculation of *p* values. To examine differences in predictors for physical activity and motivation for exercise at baseline and at follow-up (Table 3), binary logistic regression analyses were performed. In both models, the same independent variables were used. For all tests, we defined a significance level of *p*<0.05. Calculation of optimal sample size was performed by using G*Power3 [40]. Regarding the outcome variables fatigue and depressiveness at T0/T1, assuming a small effect size according to Cohen [41] and a power of *β*=0.8, a sample size of *N*=33 was determined.

**Table 1:** Patient characteristics

Characteristic	Baseline (T0) (*n=*63)	12-Months Follow up (T1) (*n*=63)	*p-*value
Gender (%)		*	
Female	41 (65.1)		
Male	22 (34.9)		
Mean age (SD; range), years	58.9 (±10.2; 34-80)	*	
Type of cancer (%)		*	
Gastrointestinal Cancer	20 (31.7)		
Breast Cancer	18 (28.9)		
Lung Cancer	15 (23.8)		
Others ^a^	5 (7.9)		
Head and Neck Cancer	3 (4.8)		
Sarcoma	2 (3.2)		
Cancer therapy ^b^ (%)			0.716 ^f^
Chemotherapy	35 (55.6)	32 (50.8)	
Immunotherapy	16 (25.4)	15 (23.8)	
Targeted therapy	11 (17.5)	12 (19.0)	
Combination w/ Hormonal treatment	7 (11.1)	3 (4.8)	
Monotherapy	4 (6.3)	9 (12.3)	
Antihormonal treatment	1 (1.6)	4 (6.3)	
Previous palliative chemotherapy (%)	36 (57.1)	*	
Number of lines, mean (SD; range)	1.1 (±1.4; 0-7)	2.0 (±1.8; 0-9)	<0.001
ECOG-status (%)			0.548 ^f^
0	32 (50.8)	24 (38.1)	
I	25 (39.7)	21 (33.3)	
II	3 (4.8)	3 (4.8)	
III	0 (0)	2 (3.2)	
ECOG < 2	57 (90.5)	45 (71.4)	0.011 ^f^
Missing	3 (4.8)	10 (15.9)	
Comorbidities (%)		*	
Cardiovascular disease	35 (55.6)		
Anemia ^c^	12 (19.0)		
Orthopaedic illness ^d^	15 (23.8)		
Thyroid gland disease	11 (17.5)		
Pulmonary disease	11 (17.5)		
Diabetes mellitus	8 (12.7)		
Psychiatric disease	12 (19.0)		
Polyneuropathy	7 (11.1)		
Number of comorbidities, mean (SD, range)	2.5 (±1.7; 0-7)	*	
Self-assessed Physical Activity (%) ^e^	14 (22.2)	16 (25.4)	0.835 ^f^
Self-reported Motivation	1.31 (±1.3; 0-4)	1.22 (±1.3, 0-4)	0.710 ^g^
Cancer-related Fatigue (FACT-F Score), mean (SD, range)	29.1 (± 9.3; 9.8-45.0)	25.6 (± 10.9; 3.0-50.0)	0.017 ^g^
Depressiveness (PHQ8-Score), mean (SD, range)	8.3 (±4.1; 1-19)	9.2 (±4.7; 1-22)	0.015 ^g^

Information is based on data of patient population at baseline (T0) and after 12 Months (T1)

Abbreviations: *ECOG*, Eastern Co-operative Oncology Group performance index; *SD*, standard; *FACT-F*, Functional Assessment of Cancer Therapy Fatigue; *PHQ8*, Patient Health Questionnaire depression scale

^a^Genitourinary Cancer, other Gynaecologic Cancers, CUP, Glioblastoma, Others

^b^Last therapy before answering the Questionnaire

^c^Haemoglobin level < 10.0 mg/dl was set for definition of “Anemia”

^d^Present orthopaedic illnesses were Arthrosis, Osteoporosis, Joint infection, Bechterews disease, Chronical Pain Syndrome, Herniated Disc, Rheumatoid Arthritis

^e^Patients claimed to be physically active at least once a weak with low intensity

^f^Fisher´s exact Test

^g^Non-parametric Wilcoxon-Test

^*^Variable was only measured at baseline (T0)

**Table 2:** Comparison of patient reported outcomes in barriers towards physical activity at baseline (T0) and after 12 months (T1)

	T0 (%) *(n*=63*)*	T1 (%) (n=63)	RR (95% CI)	*P-*value
Patient Reported Outcomes				
Sleep disturbance	51 (81.0)	46 (73.0)	1.24 (0.85–1.79)	0.398
Feeling weakened due to tumor therapy Missing	45 (71.4) 2 (3.2)	51 (81.0) 0 (0.0)	1.24 (0.78–1.98)	0.262
Lack of motivation for physical activity^b^ Missing	34 (54.0)	39 (63.9) 2 (3.2)	1.28 (0.83–1.96)	0.279
Weakness/tiredness	33 (52.4)	39 (61.9)	1.22 (0.85–1.76)	0.368
Lack of interest in exercise program^b^ Missing	30 (47.6)	31 (50.8) 3 (4.8)	1.09 (0.76–1.56)	0.645
Dyspnea	24 (38.1)	29 (46.0)	1.18 (0.8–1.67)	0.471
Deficiency in awareness of physical activity and QoL	19 (30.2)	22 (36.1)	1.14 (0.79–1.65)	0.568
Pain	18 (28.6)	26 (41.3)	1.31 (0.93–1.85)	0.191
Joint complaints	18 (28.6)	21 (33.3)	1.12 (0.78–1.60)	0.700
Fear of receiving injuries due to exercise Missing	16 (25.4) 4 (6.4)	23 (36.5)	1.22 (0.87–1.73)	0.332
Nausea/vomiting	10 (15.9)	10 (15.9)	1.00 (0.62–1.61)	1.000

^a^“Physically active”: patients were physically active at least once a weak with light intensity

^b^The 5-point Likert scale that was used during the survey was divided in two parts: “not at all”/”a little bit” were summarized as “no”; “somewhat”/”quite a bit”/”very much” were summarized as “yes”

Abbreviations: *CI*, confidence interval; *QoL*, Quality of Life; *RR*, relative risk

**Table 3:** Predictors of physical activity at baseline (T0) and after 12 months (T1)

Dependent variable: Physical activity					
	Quality of regression model	Significant predictors	Beta	*p-*value	Standard error
T0	R^2^= 0.719,	1. Dyspnoea ^b^	−6.558	0.016	2.733
	f=1.60^a^	2. Breast cancer	5.345	0.018	2.269
	χ^2^= 36.541	3. Clinically relevant depression	−3.187	0.044	1.581
	*p*<0.001	4. Motivation for physical activity	2.152	0.017	0.902
T1	R^2^= 0.704, f= 1.54^a^	1. Clinically relevant depression	−3.521	0.041	1.719
	χ^2^= 39.185 *p*<0.001	2. Motivation for physical activity	2.264	0.009	0.867

Independent variables in the regression models: clinically relevant fatigue c, knowledge about the positive effect of physical activity on quality of life, fear of receiving injuries due to exercise, sleep disturbance, weakness, feeling weakened due to active systemic cancer therapy, weakness, pain

^a^R^2^= Explained variance, f= Effect size according to Cohen

^b^PHQ8-score ≥ 10 points indicates the presence of clinically relevant depression [34]

^c^Cut-off for diagnosis of fatigue: FACT-F score ≥ 34 [31]

## Results

During the recruitment period from May 2017 to August 2018, *N*=1362 patients completed the symptom-assessment via MIDOS II [30]. Of those, *n*=725 (53.3%) questionnaires indicated moderate to severe tiredness and/or weakness. After identification of duplicates (*n*=285), 440 patients were eligible for study participation [14]. One hundred forty-one patients gave consent and answered the baseline questionnaire. Follow-up was answered by 63 participants (response rate: 44.7%) (Fig. 1). Patients who answered the questionnaire at both requested time points form in the final study cohort were referred to as “follow-up group.” Assessing the group of participants, which did not complete the follow-up (drop-out group), 46 (59.0%) patients deceased within 1 year, eight (10.3%) patients were lost, and 24 (30.8%) individuals did not answer the survey.

### Patient population

Characteristics of follow-up group at baseline and 12 months questionnaire are presented in Table 1. At baseline, 14 patients classified themselves as being physically active. After 12 months, this number increased to 16 participants. In addition to the information in Table 1, the follow-up group (*n*=63) and deceased patients (*n*=46) were compared regarding physical activity patterns, symptom burden of CRF and depression, mentioned barriers towards physical activity, and cancer-related parameters. Deceased patients reported significantly more weakness (follow-up patients, *n*=33 (52.4%); deceased patients, *n*=34 (71.7%); *p*=0.029). The follow-up group had a better ECOG performance status (*p*=0.049) and less patients received chemotherapy (follow-up group, *n*=35 (55.6%); deceased patients, *n*=36 (78.3%); *p*=0.016). No further differences were detected. Mean time before death of deceased patients was 6.22 (±3.3, 1–12) months.

### Patient reported outcomes: barriers towards exercise at baseline and after 12 months

Compared to baseline, two more patients claimed to be physically active at follow-up [T0, *n*=14 (22.2%); T1, *n*=16 (25.4%) [RR 1.04; 95 %; CI 0.86–1.27]). Patient-reported barriers towards physical activity at baseline and after 12 months are presented in Table 2. At baseline “sleep disturbance” was the most frequent reported barrier (*n*=51, 81.0%). “Feeling weakened due to cancer therapy” was most commonly chosen barrier in follow-up (*n*=51, 81%). Calculation of relative risk showed improvement in records of all regarded physical barriers (except prevalence of “sleep disturbance” and “nausea/vomiting”). Group comparisons between baseline and follow-up revealed no statistically significant differences between the listed barriers. At baseline, patients chose 0.27 out of the eight provided social barriers. The follow-up revealed a mean of 0.34 in the chosen number of social barriers. Neither comparisons in mean (*p=*0.828) nor group comparisons concerning each social barrier (except for “no local physiotherapist” [RR 2.15 95 %; CI 1.77–2.60; *p*=0.006]) showed remarkable discrepancies between the two dates of questionnaire. After 12 months, fewer patients had an ECOG performance status of 0 or I [RR 1.27 95%CI 1.06–1.51; *p*=0.011]. Mean number of palliative chemotherapy line was significantly higher at T1 (T0, 1.1(**±** 1.4); T1, 2.0 (±1.8); *p*<0.001)

### Changes of means in fatigue, depressiveness, and self-reported motivation

Comparisons in means regarding fatigue at baseline (T0) and after 12 months of cancer treatment (T1) are reported in Fig. 2a. A significant decrease of FACT-F score (3.5 points on average*, p=*0.017) among the study population could be detected. Subgroup analyses in means of FACT-F score showed a significant improvement in patients physical inactivity (3.9 points on average; *p*=0.007), while means of FACT-F score did not change significantly in self-assessed physically active participants (0.3 points on average, *p=*0.836). There were no significant differences in numbers of patients diagnosed with clinically relevant fatigue (pathological FACT-F score; ≤ 34 points [34]; T0, *n*= 41, 65.1%; T1, *n*=50, 79.4%; *p=*0.111). In the subgroup of physically inactive patients, substantially more participants suffered from clinically relevant fatigue (T0, *n*=30, 47.6%; T1, *n*=38, 60.3%; *p=*0.044). As presented in Fig. 2b, PHQ8 scores raised significantly (0.9 points on average, *p*=0.015) within ACP. Physically inactive participants had higher depression score at T1 (1.5 points on average, *p*=0.011). Physically active participants did not show any increase in PHQ8 scores (0.0 points on average, *p*=0.799). After 12 months of treatment, more patients could be diagnosed with clinically relevant depression [36] (PHQ8≥10; T0, *n*=19, 30.2%; T1, *n*=32, 50.8%; *p=* 0.029).

No statistically relevant increase or decrease could be identified in the patients’ self-reported motivation towards physical activity. Neither physically active nor physically inactive individuals had alterations relating to this variable. Physically active patients claimed to be more motivated for physical activity at any time (T0, *p<*0.001; T1, *p<*0.001).

### Prediction of subjective physical activity

Binary regression analyses were performed in order to determine predictors of patient-reported physical activity at T0 and T1 (Table 3). Significant parameter regarding physical activity in physically active/inactive patients was included in the analysis. Both models were significant and showed strong goodness-of-fit (T0, *R*^2^= 0.719, *f*=1.60; T1, *R*^2^= 0.704, *f*= 1.54) according to Cohen [41]. The model demonstrated that 71.9% (T0) and 70.4% (T1) of physically active behavior could be explained by the independent variables. *Motivation for physical activity* (T0, *β=*2,152, *p*=0.017; T1, *β*=2.264, *p*=0.009) and *clinically relevant depression* (T0, *β*=−3.187 *p*=0.044; T1, β=−3.521, *p*=0.041) were significant predictors for physical activity at both time points. At baseline, *breast cancer* (*β*=5.345, *p=*0.018) and *dyspnea* (*β*=−6.558, *p*=0.016) were further identified predictors.

## Discussion

This study aimed to reevaluate ACPs’ expression of fatigue, depression, and motivation and barriers for exercise after 12 months of cancer treatment. Possible changes in predictors of being physically active were determined by using logistic regression models.

Although patients were in an early stage of their palliative trajectory and over 90% had a good ECOG performance status, only one-fifth were physically active. Only two more participants engaged in physical activity at T1. Though reported barriers of physical activity did not increase in statistically significant value after 12 months, an increase in almost every reported barrier (except for sleep disturbance) was evident. Feeling weakened due to active systematic cancer treatment increased by 10% during 1 year of cancer treatment. The stated results also agree with the increase of ECOG and number of palliative chemotherapy lines. Various reasons can lead to this deterioration in treatment of ACP (e.g., not enough food intake, anorexia, reduced physical activity, tumor interaction). Additionally the direct influence of the palliative chemotherapy affecting different cytokines, lipolysis, proteolysis, and metabolism leads to adipose tissue and skeletal muscle mass loss [42]. Pathomechanisms may result in weakness and reduced physical functionality during advanced cancer treatment. Solheim et al. [43] measured change in weight, muscle mass, physical activity, and survival as secondary outcome of a multimodal exercise intervention in patients with incurable pancreatic and lung cancer suffering from cachexia. Though there were no significant differences between intervention and control group, muscle mass and weight remained stable among the intervention group. Muscle mass and weight remained stable among the intervention group. These results indicate that clinical deterioration of ACP could be slowed by multimodal interventions including exercise. Therefore, physical performance status should be evaluated on a regular basis during treatment of ACP. Standardized assessments of submaximal cardiorespiratory fitness and functional mobility might help to tailor physical activity programs to ACPs’ needs and abilities.

Our investigation is one of the studies that examined exercise in ACP with clinical depression as secondary outcome. Cormie et al. [44] and Tsianakas et al. [45] analyzed the effects of resistance and walking exercise in ACP on depression but did not find any significant results. Depression score stayed stable within little fluctuations. By contrast Pyszora et al. [11] presented significant findings related to decreased depression after a 2-week intervention of 30 min active exercise, myofascial release, and proprioceptive neuromuscular facilitation. Literature that highlights the influence of exercise on physical function/performance status, psychosocial symptoms, and quality of life in ACP are necessary in order to evolve solutions. Furthermore, Nipp et al. [46] demonstrated that the ongoing loss of weight/muscle mass and impairment in physical function is associated with a higher clinical depression.

The performed comparisons in mean of FACT-F score at T1 and T0 revealed an increase of FACT-F of 3.5 points on average. This points to a clinically relevant improvement of fatigue among our study cohort [31]. Only few studies have examined the course of fatigue during treatment of ACP and obtained different results. In 2016 Peters et al. [25] published a study that assessed the severity of CRF in patients undergoing palliative care over a mean time of 4.9 months. Fatigue remained steady among the population. Results of a prospective investigation by Verkissen et al. [29] showed that most symptoms including fatigue did not change significantly during treatment of ACP. In a cross-sectional study by Beernaert et al. [47], QoL was assessed in patients at three different treatment phases (curative, life-prolonging, and highly advanced). Participants in further stages of cancer treatment showed higher symptom burdens (especially fatigue) and lower QoL. In order to explain this deviation in findings, further longitudinal studies are required.

Currently, the impact of (patient-reported) physical activity on CRF in ACP is a highly discussed topic. The results of our non-interventional study suggest that CRF does not increase in patients that classified themselves as physically active. Several studies emphasized the positive effects of exercise on fatigue, QoL, and physical functioning [8, 9, 11]. A systematic review of physical activity interventions in ACP described contradictory results regarding the outcome of CRF [48]. The small sample size of our study could have lowered the power to identify significant differences in fatigue of physically active patients. In addition, our physical activity assessment grounded on subjective self-rated physical activity of our participants. Therefore, our results cannot be generalized, and more data on physical activity and CRF in ACP is necessary. Lately, Poort et al. [12] demonstrated in a randomized controlled trial that cognitive behavioral therapy improved physical functioning, QoL, and fatigue in a sample of ACP, but no statistically relevant alterations in patients receiving graded exercise therapy were detected. Referring to the close relationship of effective factors and physical activity (mentioned above), these two therapy approaches might reinforce each other as part of interdisciplinary programs.

The conducted analyses identified patient-reported *motivation for physical activity* and *clinically relevant depression* [36] as significant predictors for physical activity at both time points of survey. While motivation for physical activity was positively associated, depressiveness turned out to be a negative predictor. Baseline values of dyspnea (negative impact) and the tumor entity of breast cancer (positive impact) were substantial predictors for physical activity, which diminished over time. Some studies have examined predictors of this outcome in advanced cancer patients. Only few of them analyzed the course of predictors during cancer treatment. Ungar et al. [49] investigated physical activity enjoyment and self-efficacy in a mixed population of cancer survivors and ACP before and after a 4-week intervention and 10 weeks later. Self-efficacy and physical activity enjoyment at T0 were significantly associated with physical activity, whereas 10 weeks later only self-efficacy remained a considerable predictor. A systematic review of Ormel et al. [50] summarized predictors of adherence to physical activity in patients during and after cancer treatment. High motivation, high self-efficacy, and extensive exercise history correlated with better adherence to exercise programs. These findings indicate that performing physical activity strongly depends on patients’ psychological conditions such as motivation, self-efficacy, and depressiveness. Therefore, psychological counseling might be a promising way to promote physical activity in this particular patient population.

## Limitations

There are several limitations to our study, which should be acknowledged. We measured the participants’ subjective perception of physical activity levels, and reports might differ from objectively assessed physical activity levels. Second, participants’ answers to the related questions were individual, and a generalization of our results is not possible. In order to develop suitable activity programs, we focused on participants’ attitude towards physical activity and its surrounding aspects. This inevitably includes their subjective opinions. This study had a monocentric setting and was performed in an outpatient care of a sizeable oncologic center in Germany. The different diagnoses among our study population may not be representative. The sample size of our study was relatively small. Therefore, our results only show tendencies, and more longitudinal analyses are required. Additionally, it should be considered that most of our participants were in an early stage of their disease. The majority of our cohort had a good performance status, and approximately 40% did not have palliative chemotherapy previous to answering the baseline questionnaire.

## Conclusion

Cancer-related fatigue and depression increased in a clinically relevant dimension over the period of a year. Patients that rated themselves as physically active did not show significant progress in these symptoms. A motivated attitude and clinically relevant depression were identified as long-term predictors of subjective physical activity. Physical barriers were stated frequently but stayed stable at both measurements. Our findings emphasize the importance of psychological conditions and effective factors in physical activity behavior of ACP. Our results are in line with the latest interventional studies [12], highlighting that treatment programs for CRF should focus on early integration of both physical activity and psychological well-being. Interdisciplinary care programs that unite these two therapy concepts might help ACP not only in starting and maintaining physical activity but also in improving their psychological state of health. A sustainable decrease of fatigue and increase of patients’ QoL might be the promising outcome of this therapeutic approach.
